# An N-glycoproteomic site-mapping analysis reveals glycoprotein alterations in esophageal squamous cell carcinoma

**DOI:** 10.1186/s12967-022-03489-2

**Published:** 2022-06-25

**Authors:** Yingzhen Gao, Liuyi Shen, Tianyue Dong, Xin Yang, Heyang Cui, Yanlin Guo, Yanchun Ma, Pengzhou Kong, Xiaolong Cheng, Ling Zhang, Yongping Cui

**Affiliations:** 1 Key Laboratory of Cellular Physiology of the Ministry of Education, Department of Pathology, Shanxi Medical University, Taiyuan, Shanxi 030001 People’s Republic of China; 2 Cancer Institute, Peking University Shenzhen Hospital, Shenzhen Peking University-the Hong Kong University of Science and Technology (PKU-HKUST) Medical Center, Shenzhen, 518035 People’s Republic of China

**Keywords:** ESCC, Glycoprotein, N-glycosylation, N-glycoproteomics, Lymph node metastasis

## Abstract

**Background:**

Aberrant glycosylation has been recognized as a hallmark of cancer and N-glycosylation is one of the main types of glycosylation in eukaryotes. Although N-glycoproteomics has made contributions to the discovery of biomarkers in a variety of cancers, less is known about the abnormal glycosylation signatures in esophageal squamous cell carcinoma (ESCC).

**Methods:**

In this study, we reported the proteomics and N-glycoproteomic site-mapping analysis of eight pairs of ESCC tissues and adjacent normal tissues. With zic-HILIC enrichment, TMT-based isobaric labeling, LC–MS/MS analysis, differentially expressed N-glycosylation was quantitatively characterized. Lectin affinity enrichment combined with western blot was used to validate the potential biomarkers in ESCC.

**Results:**

A series of differentially expressed glycoproteins (e.g., LAMP2, PLOD2) and enriched signaling pathways (e.g., metabolism-related pathway, ECM-receptor interaction, focal adhesion) were identified. Besides that, seven significantly enriched motifs were found from the identified N-glycosylation sites. Three clusters were identified after conducting the dynamic profiling analysis of glycoprotein change during lymph node metastasis progression. Further validation found that the elevated fucosylation level of ITGB1, CD276 contributed to the occurrence and development of ESCC, which might be the potential biomarkers in ESCC.

**Conclusion:**

In summary, we characterized the N-glycosylation and N-glycoprotein alterations associated with ESCC. The typical changes in glycoprotein expression and glycosylation occupancy identified in our study will not only be used as ESCC biomarkers but also improve the understanding of ESCC biology.

**Supplementary Information:**

The online version contains supplementary material available at 10.1186/s12967-022-03489-2.

## Introduction

Esophageal cancer is one of the most common malignant tumors worldwide. Around 50% of global esophageal cancer patients are in China, where the main histologic type is esophageal squamous cell carcinoma (ESCC). ESCC patients tend to have a very poor prognosis with the 5-year survival rate less than 30% [1]. Although the combined usage of some markers such as squamous cell carcinoma antigen (SCC), carcinoembryonic antigen (CEA) and cytokeratin-19-fragment (CYFRA21-1) is helpful for the diagnosis and prognosis prediction of ESCC patients [2], limited markers are used in the early diagnosis of ESCC. Radical surgery is usually considered as a better option for clinical treatment. However, most ESCC patients are in the middle or late stages at initial diagnosis, and the recurrence remains a clinical problem for them. Although some drugs such as trastuzumab and ramucirumab have been applied in clinic, there is still a lack of specific and effective therapeutic targets in ESCC compared with other cancer types [3].

Aberrant glycosylation has been recognized as one of the hallmarks of cancer, which confers a new perspective for cancer research [4]. As a prominent modification type of proteins, glycosylation plays key roles in a variety of biological process such as cell transformation, differentiation, growth, invasion, and immune surveillance of tumors [5]. The most common type of glycosylation is N-linked glycosylation, occurring with the addition of specific glycan residues to asparagine [5].

More than half of cancer biomarkers are glycosylated proteins, which serve as biomarkers for either the early detection or the efficacy evaluation of treatment and prognosis. Therefore, glycoproteomics has become a popular field that can make contributions to the discovery of biomarkers of diseases. So far, glycoproteomic analysis of cell line, tissues and body fluids have shown great promise for the purpose in a variety of cancers including pancreatic cancer, prostate cancer, breast cancer, colorectal cancer, ovarian cancer and so on [6–13]. Except for some classical glycoproteins (e.g., CA125 for ovarian cancer, AFP for liver cancer and CEA for colon cancer) or glycan-related proteins (e.g., CA19-9 for gastrointestinal and pancreatic cancer) frequently used in clinical practice, these studies also identified some new glycoproteins that might be potential promising biomarkers in cancers [14]. For example, fucosylated haptoglobin (Fut-Hpt) can be used as a biomarker for liver metastasis of colorectal and pancreatic cancer [15]. Moreover, antibodies of the fucosylated Le Y have also been used as potential drugs for immunotherapy in epithelial-derived tumors [16]. Glycoproteomics identified HOMER3 as a potentially targetable biomarker triggered by hypoxia and glucose deprivation in bladder cancer [17]. The glycoproteomics in high-grade serous ovarian carcinoma delineated three major clusters, showing a strong relationship between fucosylation and mesenchymal subtype, which predicted a poor prognosis for cancer patient [18]. Thus, further investigation of the glycosylation changes in cancer especially ESCC may provide a new way for clinical application.

Abnormal glycosylation usually includes the changes of glycoprotein expression level caused by abnormal glycosylation modification and changes of glycan structure of glycoproteins determined by glycogene alteration. However, the complex characteristics of glycans and the limitation of research methodology make glycoproteomics and glycomics study severely lag behind. To date, less is known about the abnormal glycosylation signatures in ESCC. Here, we performed N-glycoproteomic site-mapping analysis to investigate the differentially expressed glycoproteins in ESCC. Since the changes of glycoproteins may be caused by the variation of protein concentration [19], we integrated proteomics and N-glycoproteomics data for protein normalization of the identified glycosylation changes. Moreover, we investigated dynamic profiling changes in glycoprotein during lymph node metastasis progression and validated some potential biomarkers in ESCC. This study might throw new light on the early diagnosis and molecular targeted treatment of ESCC.

## Materials and methods

### Patient tissue samples

The eight pairs of tumor tissues and adjacent normal tissues at least 5 cm from the center of the tumor used for N-glycoproteomic site-mapping and proteomics analysis in this study were obtained from patients diagnosed with ESCC who underwent surgical resection at the Shanxi Cancer Hospital (Taiyuan, China). The five pairs of tissues used in UEA-I lectin affinity chromatography were obtained from the same source as above. There was no preoperative chemotherapy, radiotherapy or other therapies done on these patients. All the recruited patients gave their informed consent before they participated in our study. The study was conducted in accordance with the Declaration of Helsinki, and the protocol was approved by the Ethics Committee of the Shanxi Medical University (Approval No. 2017LL037). Details of clinicopathological features of cases are summarized in Additional file 1: Table S1.

### Protein extraction and trypsin digestion

After the samples were ground into cell powder in liquid nitrogen, four times the volume of lysis buffer (1% protease inhibitor, 3 μM TSA, 1% TritonX-100, 10 mM dithiothreitol, 50 mM NAM and 2 mM EDTA) was added and the samples were sonicated on ice using an ultrasonic processor. An equal volume of Tris-saturated phenol (pH 8.0) was added. Then, the mixture was further vortexed for 5 min. After centrifugation (4 °C, 10 min, 5,000 g), the upper phenol phase was transferred to a new centrifuge tube. Proteins were precipitated by adding at least four volumes of ammonium sulfate-saturated methanol and incubated at − 20 °C for at least 6 h. After centrifugation at 4 °C for 10 min, the supernatant was discarded. The remaining precipitate was washed with ice-cold methanol, followed by ice-cold acetone for three times. The protein was redissolved in 8 M urea and the protein concentration was determined with BCA kit according to the manufacturer’s instructions. Then the samples were reduced with 5 mM dithiothreitol for 30 min at 56 °C, iodoacetamide was added to make the final concentration 11 mM and samples were incubated at room temperature for 15 min in darkness. Finally, the urea concentration was diluted to less than 2 M. Trypsin was added at 1:50 trypsin-to-protein mass ratio overnight for the first digestion and 1:100 trypsin-to-protein mass ratio for 4 h for the second digestion to make the digestion more sufficient.

### TMT labeling and PTM enrichment for N-glycosylation

The initial amount of each sample was 200 μg. The digested peptides were desalted using Strata X C18 SPE column (Phenomenex) and vacuum-dried. Then, the peptides were reconstituted in 0.5 M TEAB buffer and labeled with TMT kit according to the manufacturer’s protocol. Briefly, the peptides were incubated with TMT reconstituted in acetonitrile for 2 h at room temperature, then pooled, desalted and dried. The peptides were dissolved in 40 µl enrichment buffer containing 80% acetonitrile/1% trifluoroacetic acid. Then the supernatant was transferred to a hydrophilic (HILIC, Click maltose) micro-column. After centrifugation at 4,000*g* for 15 min, the HILIC micro-column was washed three times. Subsequently, glycopeptides were eluted using 10% acetonitrile and the eluate was collected. After being vacuum freeze-dried, samples were dissolved in 50 mM of ammonium bicarbonate buffer dissolved in 50 μl heavy oxygen water. Finally, 2 μl of PNGase F (1000 units) was added and samples were digested overnight at 37 °C. For LC–MS/MS analysis, the resulting peptides were desalted with C18 ZipTips (Millipore) according to the manufacturer’s instructions.

### HPLC fractionation and LC–MS/MS analysis

The tryptic peptides were fractionated into fractions by high pH reverse-phase HPLC using Agilent 300Extend C18 column (5 μm particles, 4.6 mm ID, 250 mm length). Firstly, the peptides were separated with a gradient of 8–32% acetonitrile (pH = 9.0) for more than 60 min into 60 fractions. Then, the peptides were combined into 18 fractions and dried by vacuum centrifuging. All of the above processes, LC–MS/MS and subsequent bioinformatics analyses were performed in Jingjie PTM Biolabs (Hangzhou in China). The peptides were dissolved in 0.1% formic acid (solvent A) and directly loaded into a reversed-phase analytical column (75 μm i.d, 25 cm length). The gradient consisted of a gradual increase from 5 to 20% of solvent B (0.1% formic acid in 90% acetonitrile) for more than 40 min, 20% to 35% in 12 min and climbing to 80% in 4 min, followed by being held at 80% for the last 4 min. Above all were operated at a constant flow rate of 700 nl/min on EASY-nLC 1000 UPLC system. The peptides were submitted to NSI source and analyzed by tandem mass spectrometry (MS/MS) in Orbitrap Fusion (Thermo). The m/z scan range was 350–1550 for full scan, and Orbitrap was used to detect intact peptides at a resolution of 60,000. Then, peptides were selected and analyzed by MS/MS using NCE setting as 35 and Orbitrap was used to detect the fragments at a resolution of 30,000. Automatic gain control (AGC) was set at 5E4. Fixed first mass was set as 100 m/z.

### Database search

Maxquant search engine (v1.5.2.8) was used to retrieve the mass spectrometer data. Parameter settings were as follows. The database was SwissProt Human (20,130 sequences), and the reverse decoy database was added to calculate the false positive rate (FDR) caused by random matching. A common contamination database was used to eliminate the influence of contaminated proteins. Trypsin/P was specified enzyme allowing up to 2 missing cleavages. The minimum required peptide length was set to seven amino acids and the maximum modification number of the peptide was set to 5. In first search, the mass tolerance for precursor ions was set as 20 ppm. In main search, the mass tolerance for precursor ions was set as 4.5 ppm. The mass tolerance for secondary fragment ions was set as 0.02 Da. Carbamidomethyl on Cys was specified as fixed modification. The variable modification was defined as oxidation on Met, deamidation 18O (N), deamidation (NQ), and acetylation of the N-terminal of the protein. The quantitative method was set to TMT-10plex. FDR of proteins and PSM identification was adjusted to 1% and minimum score for modified peptides was set > 40.

### Gene Ontology (GO) and KEGG pathway annotation

The annotation of Gene Ontology (GO) of proteins comes from the UniProt-GOA database (http://www.ebi.ac.uk/GOA/). Firstly, the system converted protein ID to UniProt ID, then used UniProt ID to match GO ID, and extracted corresponding information from UniProt-GOA database based on GO ID. If there was no according protein information in UniProt-GOA database, the InterProScan soft based on protein sequence would be used to annotate protein’s GO function. The proteins were then classified according to cellular component, biological process and molecular function. Kyoto Encyclopedia of Genes and Genomes (KEGG) database was used to annotate protein signaling pathway. Firstly, we used KEGG online service tools KAAS to annotate KEGG database description of proteins. Then KEGG mapper, KEGG online service tools was used to map the annotation result on the KEGG pathway database. Wolfpsort, an updated version of PSORT/PSORT II, was applied to predict subcellular localization.

### Motif analysis

Software MoMo (motif-x algorithm) was applied to analyze the motif characteristics of N-glycosylation sites. Among them, the peptide sequences of 10 amino acids upstream and downstream of all the N-glycosylation site were analyzed. And all the database protein sequences were used as background database parameter. When the number of peptides in a specific sequence was more than 20 and the p value was less than 0.00000 1, it was considered that the characteristic sequence was a motif of modified peptide. The calculation method is as follows: (the number of peptide identified with a certain motif/the number of peptide identified with Ng)/(the number of peptide identified with this motif in the database/the number of theoretical peptide in which Ng occurs in the database).

### UEA-I lectin affinity chromatography

Sufficient tissue samples were fully ground in liquid nitrogen. The protein lysate (Beyotime, #P0013) and protease inhibitors were added to the samples. The samples were incubated on ice for 1 h and centrifuged at 4 °C for 20 min. The supernatant was total protein solution and protein concentration was measured using the BCA method. 50 µg total-protein was taken as input and 2,000 µg total-protein was mixed with 100 µl biotinylated UEA-I lectin. Next, the mixture was fixed to 1 ml with Phosphate-Buffered Saline (PBS) containing 1 mM CaCl2, MgCl2 and MnCl2. After incubation overnight at 4 °C on rotating shaker, 50 µl agarose streptavidin beads (Vector laboratories, #SA-5010) were added to the mixture and continued to be incubated for 4 h. Then the beads were washed with PBST (PBS mixed with 0.05% tween-20) five times after centrifugation, protein loading was added and the samples were boiled at 100 °C for 5 min, which followed by the western blotting.

### Western blot

All protein samples were separated on 8% SDS-PAGE by electrophoresis and transferred to PVDF membranes. After being blocked with 5% skim milk for 1 h, the membranes were incubated with the primary antibodies at 4 °C overnight, and incubated with the secondary antibody at room temperature for 2 h. Antibodies used were as follows: ITGB1 (1:1,000, Proteintech, #12594-1-AP), CD276 (1:1,000, Proteintech, #66481-1-lg), and GAPDH (1:20,000, Proteintech, #60004-1-lg). IRDye 800CW conjugated goat anti-mouse (or rabbit) IgGs was used for protein detection using the Odyssey infrared imaging system. Finally, signals were digitized by ImageJ software (v1.52i).

### Soft-clustering analysis

The Mfuzz package (version 2.36.0) based on the open-source statistical language R (version 3.6.3) was used to perform soft-clustering analysis. The fuzzy c-means algorithm, one of the most widely used soft-clustering method, provided by Mfuzz package, is more noise robust and a priori pre-filtering of genes/proteins can be avoided, so it avoided the exclusion of biologically relevant genes/proteins from the data analysis [20, 21]. FCM clustering uses Euclidean distance as the distance metric, and demands two main parameters (c = number of clusters, m = fuzzification parameter). Different from k-means clustering, each element has a set of membership coefficients corresponding to the degree of being in a given cluster by FCM clustering.

For the quantitative information of N-glycosites, we removed the N-glycosites containing missing values in the raw data at first, to make sure that each gene could be quantified in each sample. The quantitative ratio of N-glycosites was calculated in each sample pair, and the quantitative ratios were log2 transformed and then z-score was normalized for each pair of sample, making the mean zero and standard deviation one. The normalized-data made sure that N-glycosites with similar temporal patterns were close in Euclidean space [22]. The transformed profiles were then clustered using the Mfuzz package. For this analysis, the optimal values of c and m were derived. Final clustering was done with the parameters c = 15 and m = 1.48. Then we screened out the groups related to the degree of LNM, set the appropriated membership coefficients to make the remaining genes in these groups had a similar trend of change (membership ≥ 0.30).

## Results

### Study design and global proteomics analysis of ESCC

**Fig. 1:**
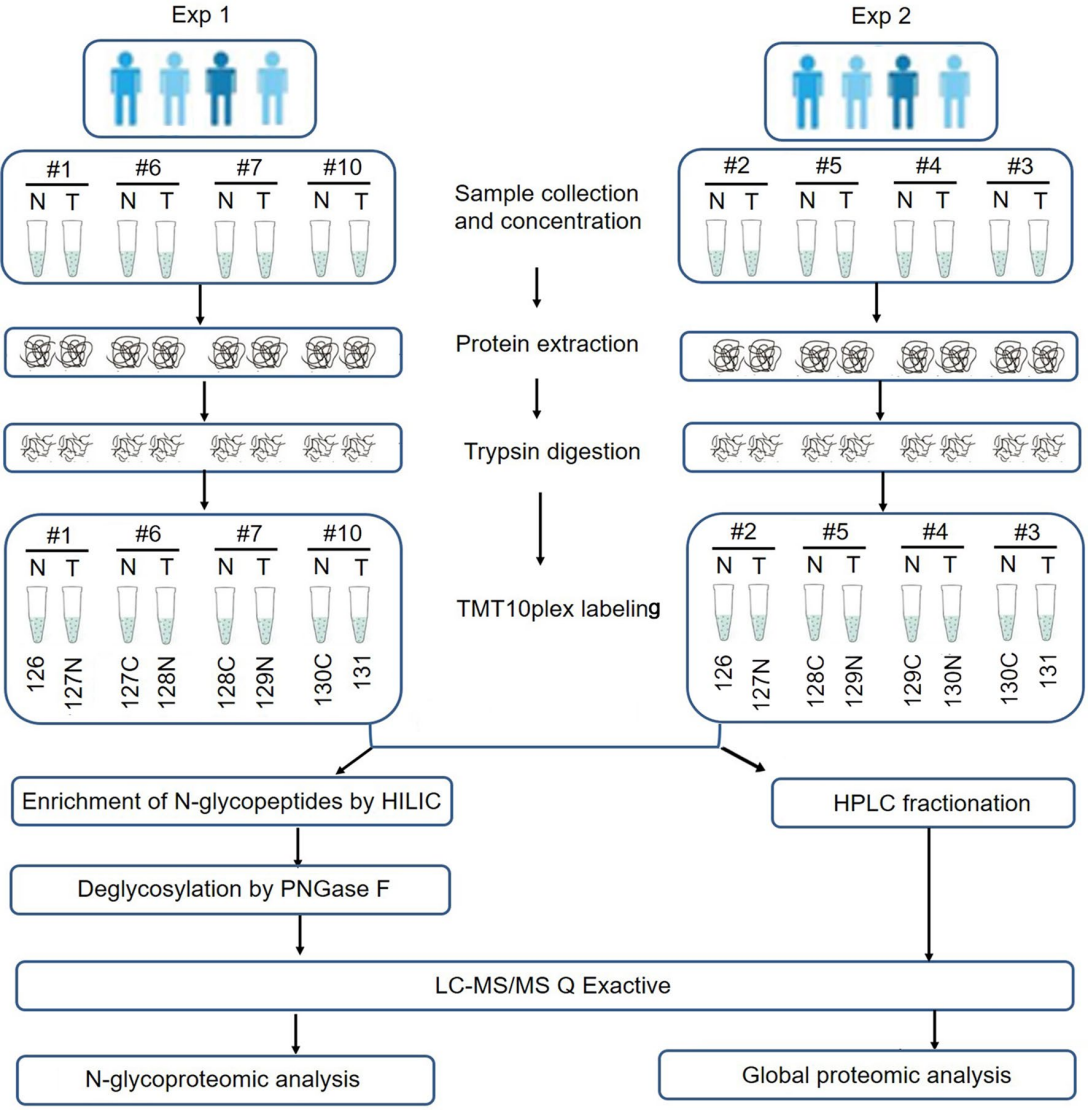
Schematic representation of the N-glycoproteomics and proteomics approach for the discovery of differential expressed glycoproteins and proteins in ESCC tissues compared with matched normal tissue

**Fig. 2:**
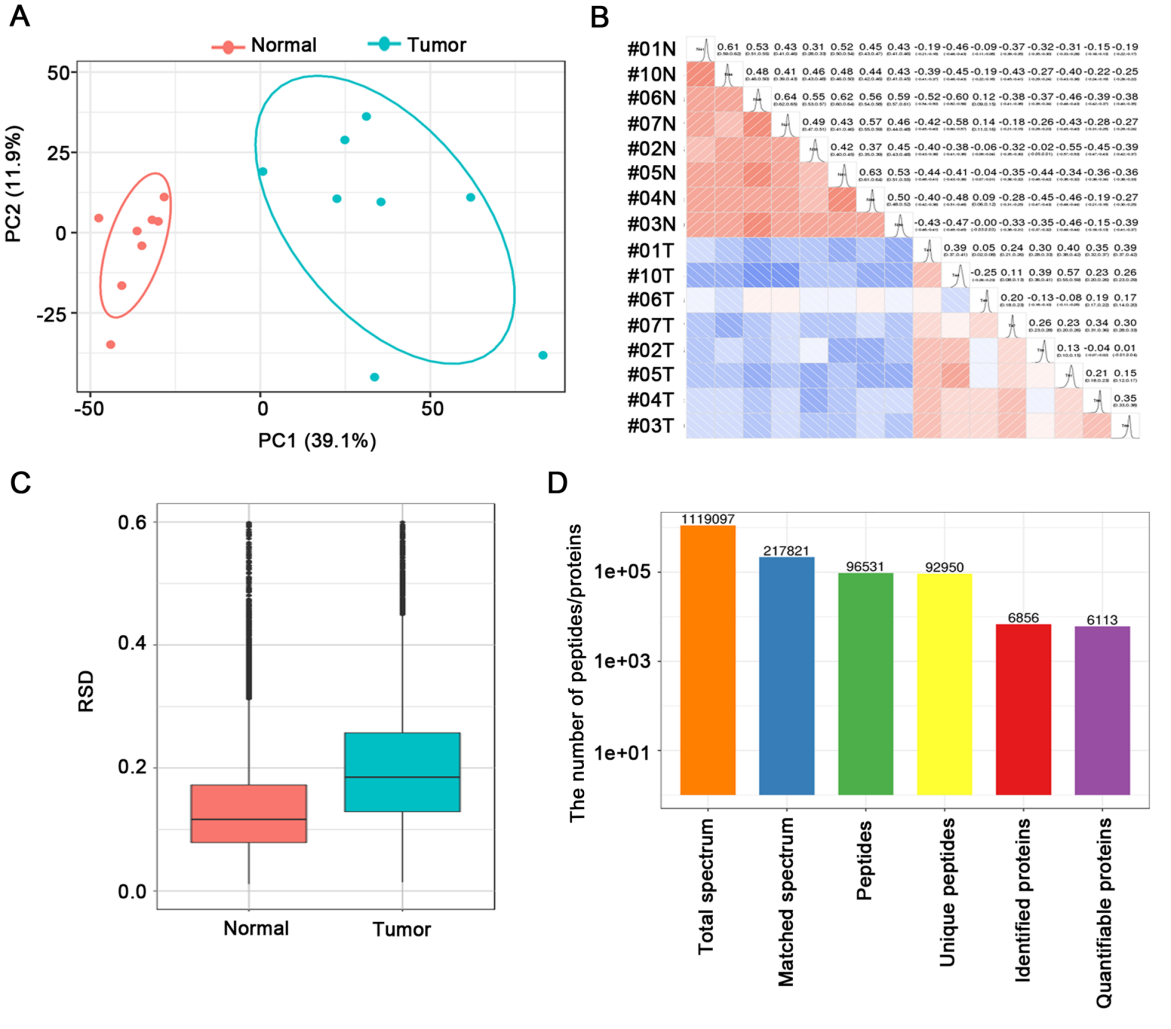
Identification of global proteins in proteomics analysis. **A** Principal Component Analysis of protein quantity in proteomics data. **B** Pearson correlation coefficient heat map of protein quantification. **C** The boxplot of RSD distribution. **D** Basic statistical graph of mass spectrometry data

**Fig. 3:**
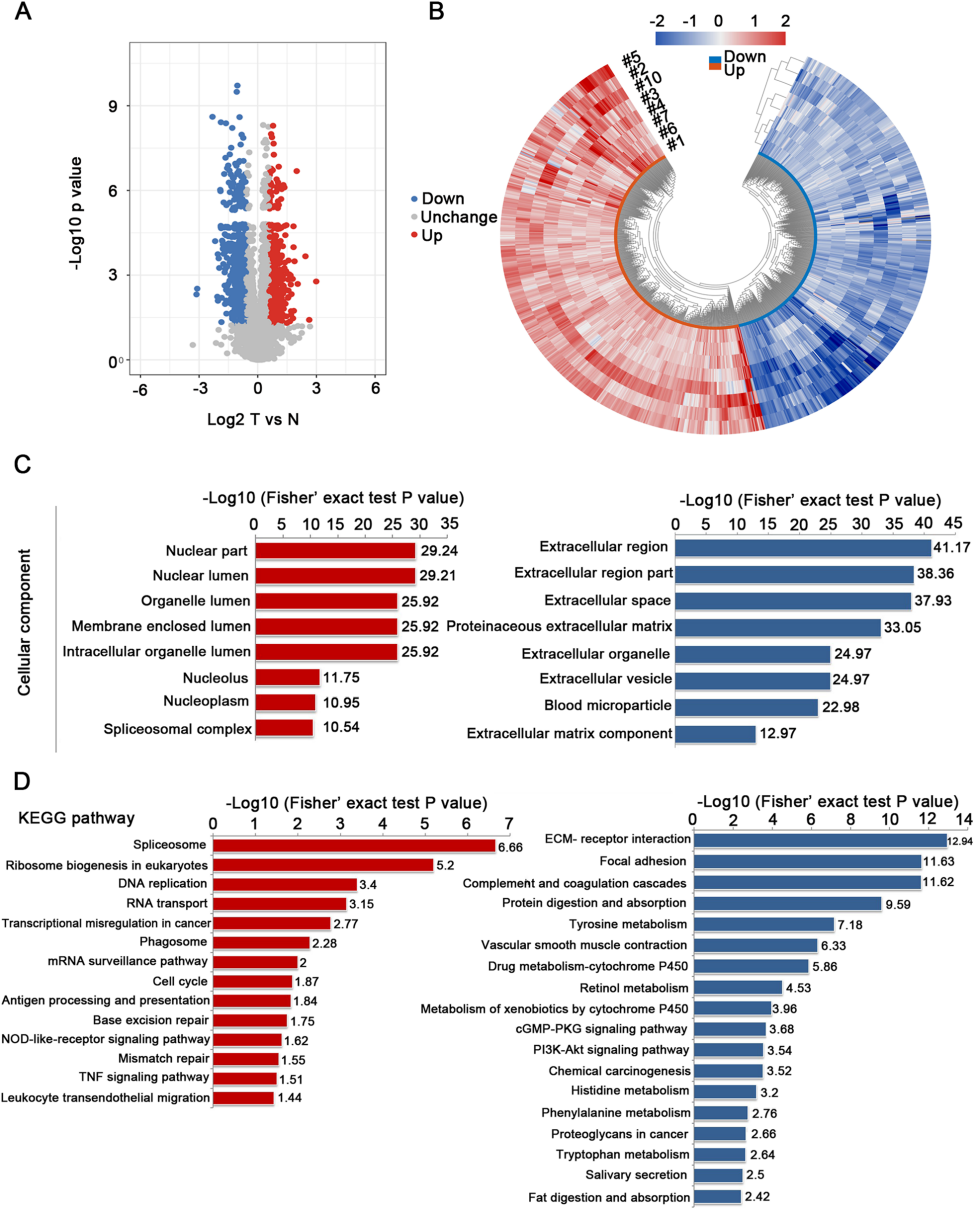
Functional analysis of global differential expressed proteins in proteomics analysis. **A** The volcano plot of differentially expressed proteins. **B** The heat map of differentially expressed protein in all paired comparisons. **C** Cellular component enrichment analysis of up-regulated (left) and down-regulated proteins (right). **D** KEGG enrichment analysis of up-regulated (left) and down-regulated proteins (right)

Figure 1 showed the workflow for the global N-glycoproteomics and proteomics analyses of the ESCC tumor and matched normal tissues. ESCC tissue and paired normal tissue samples from the same patient were used to reflect the level of different individuals. Sixteen protein samples from eight patients were assigned to two assays with TMT labeling and digested with trypsin for further analyses. In the N-glycoproteomics analysis, deglycosylated peptides after PNGase F treatment were actually measured instead. This de-N-glycoproteomics was aimed at profiling glycosylated proteins without focusing on the glycan structure, equaling to the N-glycosylation site mapping. A detailed description of the clinical characteristics of the analyzed samples was presented in Additional file 1: Table S1. To avoid unexpected changes of glycoproteins caused by the amount variation of protein samples, we firstly investigated the global proteomic profile in eight paired tumor and normal tissues using TMT labeling-based quantitative proteomic assays. Principal Component Analysis (PCA) and Pearson correlation analysis showed a clear distinction between the ESCCs and normal esophageal tissues (Fig. 2A, B). Relative standard deviation (RSD) was less than 20% in two groups, representing the ideal repeatability (Fig. 2C). The number of according peptides/proteins in basic statistical analysis of mass spectrometry was shown in Fig. 2D. These results of quality control process indicated that our proteomics analysis system was robust. Altogether, 6,856 proteins were identified from the selected samples, among which 6,113 proteins were quantified. The mean value of the 6,113 protein quantitative ratios was 1.11, indicating that there is no obvious change in protein expression levels. With fold change value > 1.5 and p-value < 0.05 as threshold, totally 596 proteins were up-regulated while 495 proteins were down-regulated (Fig. 3A). All these data were listed in Additional file 1: Table S2. Proteins showing significant up/down-regulation were described in Fig. 3B. In terms of cellular component, the up-regulated proteins were mainly localized in chromosome, nuclear lumen, spliceosomal complex and intracellular organelle lumen, while most of the down-regulated proteins were found in the extracellular region, extracellular matrix and vesicle (Fig. 3C). Accordingly, KEGG-based enrichment analysis showed that the prominent pathways enriched in the up-regulated proteins included spliceosome, DNA replication, RNA transport, transcriptional mis-regulation in cancer, cell cycle, base excision repair and mismatch repair processes. By contrast, protein expression in the cellular pathways including ECM-receptor, focal adhesion, metabolism (e.g., protein digestion and absorption; tyrosine, histidine, phenylalanine, and tryptophan metabolism; drug metabolism-cytochrome P450), interaction and proteoglycans processes was decreased (Fig. 3D). Based on the proteomics data, the following N-glycoproteomic site-mapping data was normalized by corresponding protein expression.

### Motif analysis of N-glycoproteome

**Fig. 4:**
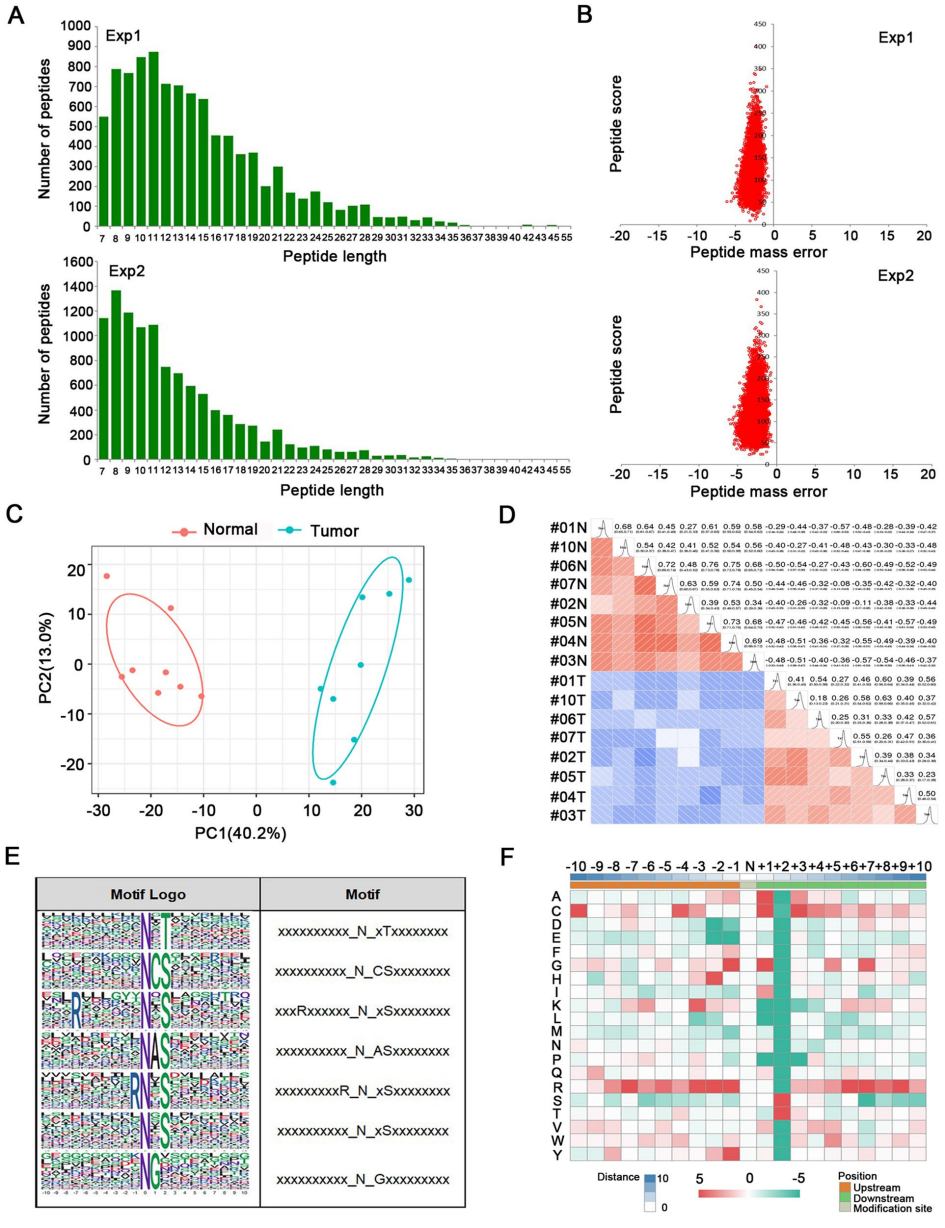
Motif analysis of the identified glycosylation peptides. **A** Statistic analysis of the whole identified peptide length in N-glycoproteomics analysis in two experiments. **B** The mass error of the whole identified peptides in N-glycoproteomics analysis. **C** Principal Component Analysis of glycoproteins quantity. **D** Pearson correlation coefficient heat map of glycoproteins quantification. **E** Sequence logo of glycosylation motifs. **F** Heatmap of amino acid frequencies of the sequences flanking N-glycosylation sites

As one of classical post-transcriptional modifications (PTM), glycosylation has been proved to be essential for regulating cellular functions. Although the N-glycoproteomics profile has been revealed in some cancer types, it remains unreported in ESCC. In this study, the quality control results of N-glycoproteomics showed that our optimized methods were highly confident. Most identified peptides were distributed in size of 8–20 amino acids, which was consistent with the common pattern after trypsin treatment (Fig. 4A). Mass error of most spectrograms was within 5 ppm, which indicated that the accuracy of the mass spectrometer was reliable and the qualitative analysis of protein would not be affected by the large mass deviation (Fig. 4B). The results exhibited reliable reproducibility and accuracy in LC–MS/MS by both Principal Component Analysis and Pearson correlation analysis (Fig. 4C, D). In total, N-glycoproteomics data quality control indicated that the analysis system was robust.

To characterize the possible specific sequence, motif analysis was performed to discover the likelihood of amino acid types being over- or under-represented surrounding the N-glycosylation sites. Seven significantly enriched motifs were identified. Motif logos according to the score ranking included N-X-T, N-C-S, R-XXXXXX-N-X-S, N-A-S, R-N-X-S, N-X-S, N-G-X (X represents a random amino acid residue; N, T, A, G, C, S, R represent asparagine, threonine, alanine, glycine, cystine, serine, arginine, respectively) (Fig. 4E). To validate whether there were position-specific amino acids surrounding N-glycosylation sites, the amino acid frequencies in the sequences flanking N-glycosylation sites were assessed using motif model (Fig. 4F). In addition to canonical N-linked glycopeptide sequon of N-X-T/S, we found that cysteine residue at + 1 and + 3, glycine residue at  − 1 and + 1, arginine residue at − 1, − 2 and − 7, and alanine residue at + 1 position were overrepresented. Interestingly, despite N-X-C and N-X-V have been discovered to be atypical N-glycosylation sequons [23], cystine and valine were found to be depleted at + 3 position of N-glycosylation site in our study. The motif score, relative frequency and other detailed information of these motif were demonstrated in Additional file 1: Table S3.

### Characteristics of the N-glycoproteins: identification and quantitative analysis

**Fig. 5:**
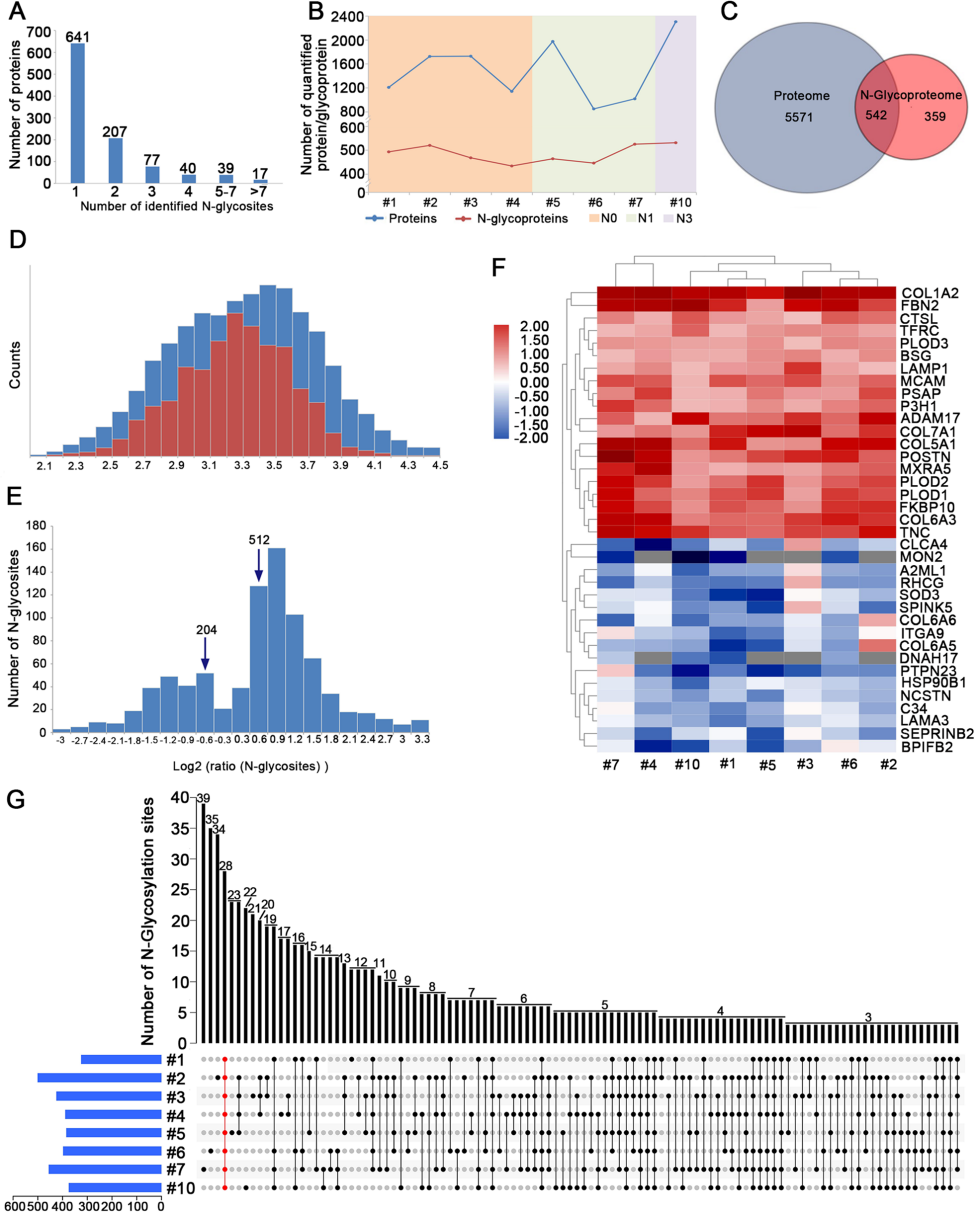
Comparative analyses of N-glycoproteomics and proteomics between ESCC and paired normal tissue. **A** Number of N-glycosylation sites identified per protein in N-glycoproteomics analysis. **B** Overview of quantified proteins and N-glycoproteins in different stage of ESCC. **C** The overlap of the proteins identified in both proteomics and N-glycoproteomics. **D** Intensity distribution of all glycosites only identified in the N-glycoproteomics data (red bars) overlaid with that of N-glycosites whose corresponding proteins were also identified in the proteomics data (blue bars). **E** Log2 ratio distributions for N-glycosites analyzed, the value greater than 0.584 or less than − 0.584 were considered to be significant. **F** Heat map of 37 common proteins that exhibited significant up-regulation in all eight comparisons and down-regulation in more than four comparisons. Color represents the expression levels of proteins, among which red color denotes the high expression while blue color denotes the low expression level. **G** Number of N-glycosylation sites identified in different samples

On average, a total of 1,839 N-glycosylation sites in 1,021 glycoproteins were identified in total, of which 1,588 sites in 901 glycoproteins were quantitative. Figure 5A demonstrated the distribution of the number of identified N-glycosylation sites in each glycoprotein. Among all identified glycoproteins, approximately 62.8% (641/1021) contained only one N-glycosylation site, 20.3% (207/1021) possessed two N-glycosylation sites, and 5.5% (56/1,021) contained more than four N-glycosylation sites. Notably, six glycoproteins were found to have more than nine N-glycosylation sites as follows, LDL receptor-related protein 1 (23 sites), laminin subunit alpha-2 (14 sites), fibrillin-1 (13 sites), laminin subunit gamma 1 (12 sites), tenascin C (10 sites) and laminin subunit alpha 5 (10 sites).

To explore the relations and difference between the global N-glycoproteins and proteins, we compared datasets of the quantified N-glycoproteomic site-mapping and the proteomics. It was demonstrated that the quantified proteins were more than glycoproteins for ESCC cases. Moreover, the numbers of quantified proteins had no trend while the numbers of quantified glycoproteins increased with the progression of lymph node metastasis (Fig. 5B). The overlapping part in Fig. 5C represents the protein groups identified in both N-glycoproteomics and proteomics. In our results, a total of 542 overlapped proteins were identified. In other words, only 60.2% (542/901) of the glycosylated proteins identified in N-glycoproteomic site-mapping analysis were also found in the proteomics analysis. In fact, some glycosylated proteins, such as surface receptors and secreted proteins, show a relatively low abundance, which makes it difficult to detect them in the proteomics analysis. This was supported by the results that the intensity of deglycoside proteins only identified in the N-glycoproteomics data was lower than that of the corresponding proteins found in the global proteomics data (Fig. 5D).

A total of 716 differentially expressed N-glycosylation sites (with a cut-off change of 1.5-fold and p-value < 0.05) in 441 proteins were characterized in our N-glycoproteomic site-mapping analysis. We found that 512 N-glycosylation sites in 326 glycoproteins were significantly up-regulated and 204 N-glycosylation sites in 115 glycoproteins were significantly down-regulated (Fig. 5E). It is well known that the glycosylation modification change may be attributed the changes of total protein expression. Thus, we mainly focused on the 542 glycoproteins that had quantitative information in both proteomics and N-glycoproteomics.

We evaluated the N-glycoproteomic ratios of 1,107 N-glycosites in 542 proteins which were normalized by their corresponding protein amount. A significant correlation between the N-glycosite ratios and protein ratios (Pearson r = 0.84) was discovered, demonstrating that the changes of N-glycosylation modification were probably caused by the protein abundance change. A total of 264 differentially expressed N-glycosylation sites in 179 proteins were identified, in which 243 N-glycosylation sites in 162 glycoproteins were significantly up-regulated and 21 N-glycosylation sites in 18 glycoproteins were significantly down-regulated. The differentially expressed glycoproteins and glycosylation sites were listed in Additional file 1: Table S4. Twenty-four glycosylation sites of 20 proteins were consistently up-regulated in all samples and nineteen glycosylation sites of 17 proteins were consistently down-regulated in more than four samples (Fig. 5F). Among them, procollagen-lysine, 2-oxoglutarate 5-dioxygenase 2 (PLOD2), collagen alpha-3 (VI) chain (COL6A3), peptidyl-prolyl cis–trans isomerase (FKBP10) and Tenascin (TNC) had two or more differential glycosylation sites in all samples. The number of differentially expressed N-glycosylation sites that were identified in individuals of both experiments was exhibited in Fig. 5G.

### Characteristics of the N-glycoproteins: functional analysis

**Fig. 6:**
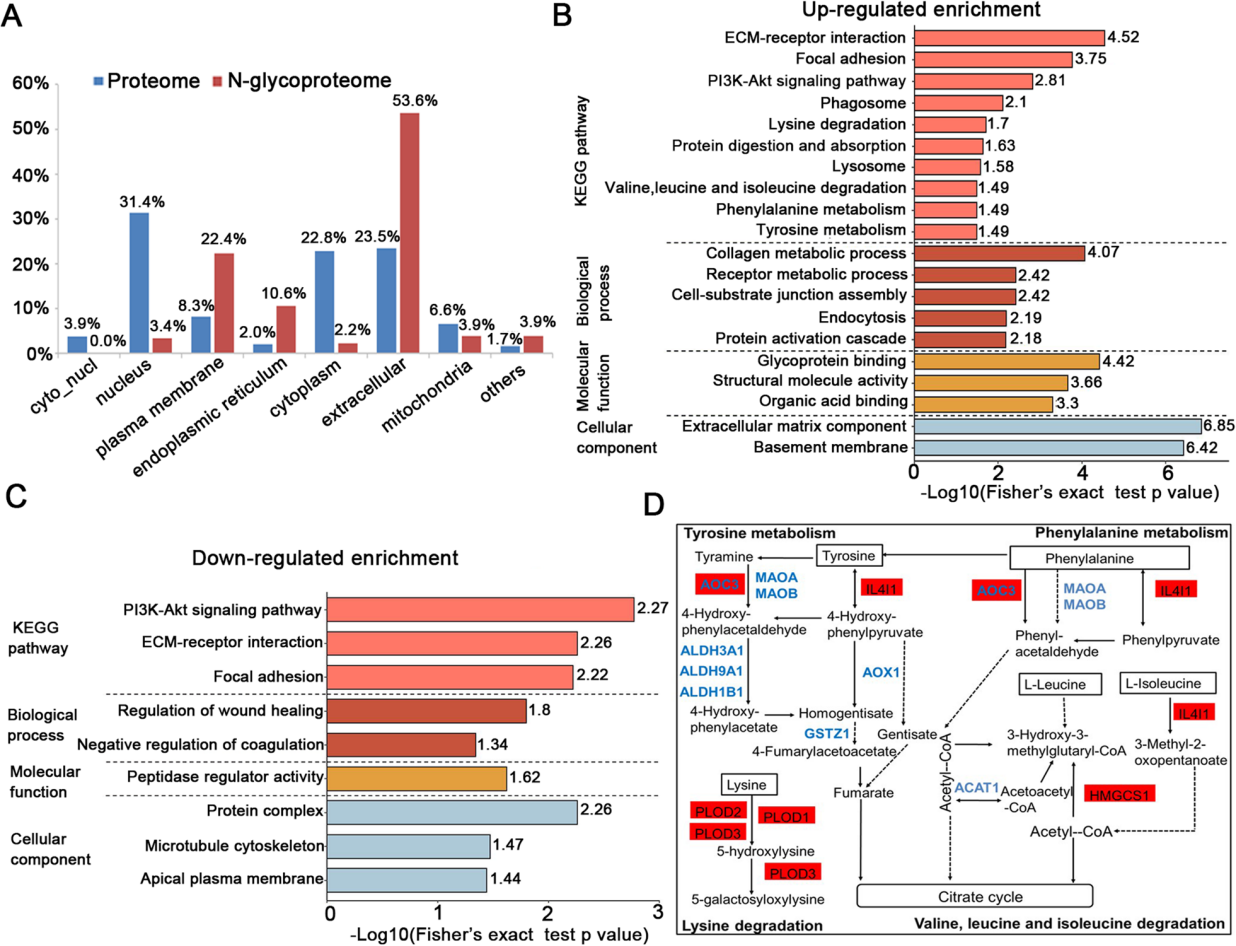
Identification and functional analysis of global normalized N-glycoproteomics in ESCC. **A** The comparison of the global proteomics and N-glycoproteomics in subcellular locations. **B** GO and KEGG enrichment analysis of up-regulated glycosylated proteins. **C** GO and KEGG enrichment analysis of down-regulated glycosylated proteins. **D** Differential expressed glycoproteins and proteins associated with amino acid metabolism-related pathways. Red box represents upregulated glycosylated proteins in N-glycoproteomics data. Blue font represents downregulated proteins in proteomics data

Subcellular localization analysis revealed that the majority of differentially expressed glycoproteins (fold change > 1.5 and p value < 0.05) distributed in the extracellular region (53.6%), plasma membrane (22.4%) and endoplasmic reticulum (10.6%). However, only a few glycoproteins were localized in the cytoplasm (2.2%) and nucleus (3.4%). It was highly consistent with the biological function of glycoproteins, completely different from the protein localization in proteomics (Fig. 6A). Then we performed enrichment analysis of the differentially expressed glycoproteins to identify the significantly Gene Ontology terms and KEGG pathways. Most up-regulated glycoproteins were enriched in cell-substrate-related metabolic processes including collagen metabolic process, receptor metabolic process, protein activation cascade. For the KEGG pathway enrichment, some known glycosylation-affected cancer-associated pathways including ECM-receptor interaction, focal adhesion, phagosome and lysosome were enriched in up-regulated glycoproteins. Interestingly, some amino acid metabolism-related pathways including lysine degradation, tyrosine metabolism, phenylalanine metabolism, valine, leucine and isoleucine degradation were glycosylated and the corresponding glycoproteins were significantly up-regulated in ESCC (Fig. 6B). Three N-glycosites (N63, N209 and N696) were identified in Procollagen-lysine, 2-oxoglutarate 5-dioxygenase 2 (PLOD2) that were reported to catalyze the hydroxylation of lysyl residues in collagen-like peptides. Other up-regulated glycoproteins related to amino acid metabolism included procollagen-lysine, 2-oxoglutarate 5-dioxygenase 1 and 3 (PLOD1 and PLOD3), amine oxidase copper containing 3 (AOC3), interleukin 4 induced 1 (IL4I1), 3-hydroxy-3-methylglutaryl-CoA synthase 1(HMGCS1) and so on. In contrast, these up-regulated pathways in N-glycoproteomic site-mapping were found to be down-regulated in proteomics, indicating these pathways can be actually regulated via glycosylation modification in ESCC. A number of the down-regulated terms were associated with peptidase regulator activity. The KEGG pathway analysis showed ECM-receptor interaction, focal adhesion and PI3K-Akt signaling pathway were negatively enriched in our results (Fig. 6C). Besides that, differential glycosylated proteins in some amino acid metabolism-related pathways were described in Fig. 6D.

### Validation of significantly altered glycoproteins between ESCC and paired normal tissues

To further identify the potential promising biomarkers in ESCC, we further validated the 179 significantly altered glycoproteins between ESCC and paired normal tissues. Firstly, 97.7% of the proteins (i.e., 175 out of 179) have been identified and validated in other proteomics studies of ESCC. Extensive literature searching uncovered evidence that 53 (i.e., 29.6%) of the 179 glycoproteins were directly related to ESCC. Moreover, literature review and glycosites database search hit 60 (33.5%) and 174 (97.2%) glycoproteins, respectively (Additional file 1: Table S5). These data at least provided the evidence that the overlapped glycoproteins probably could serve as ESCC markers.

Previously, we also systematically profiled label-free quantitative proteomes and N-glycoproteome of two ESCC cell lines. The corresponding method and quality control results were shown in the previous study [24]. After normalization by protein quantification, a total of 1,100 N-glycosylation sites in 575 glycoproteins were identified (Additional file 1: Table S6). We integrated significantly altered glycoproteins at ESCC tissue level with the identified glycoproteins at ESCC cellular level. A total of 88 glycoproteins were identified to be overlapped (Additional file 1: Table S5). The identified overlapped proteins may be truly ESCC specific.

Also, we previously used lectin affinity chromatography and LC/MS/MS analysis to elucidate the glycosylation patterns in ESCC cell lines. The lens culinaris agglutinin (LCA; mannose, glucose, core fucose-binding) and ulex europaeus agglutinin 1 (UEA-I; fucose-binding) were applied [24]. The 707 and 956 glycoproteins were identified respectively (Additional file 1: Tables S7 and S8). We performed a combined analysis of 179 significantly altered glycoproteins in this study and lectin enriched glycoproteins in the previous study. A total of 21 and 40 glycoproteins were identified to be overlapped respectively (Additional file 1: Table S5). These data perhaps shed light on the rough estimate of glycan patterns and candidate biomarkers of ESCC for distinguishing cancer patients from healthy individuals.

**Fig. 7:**
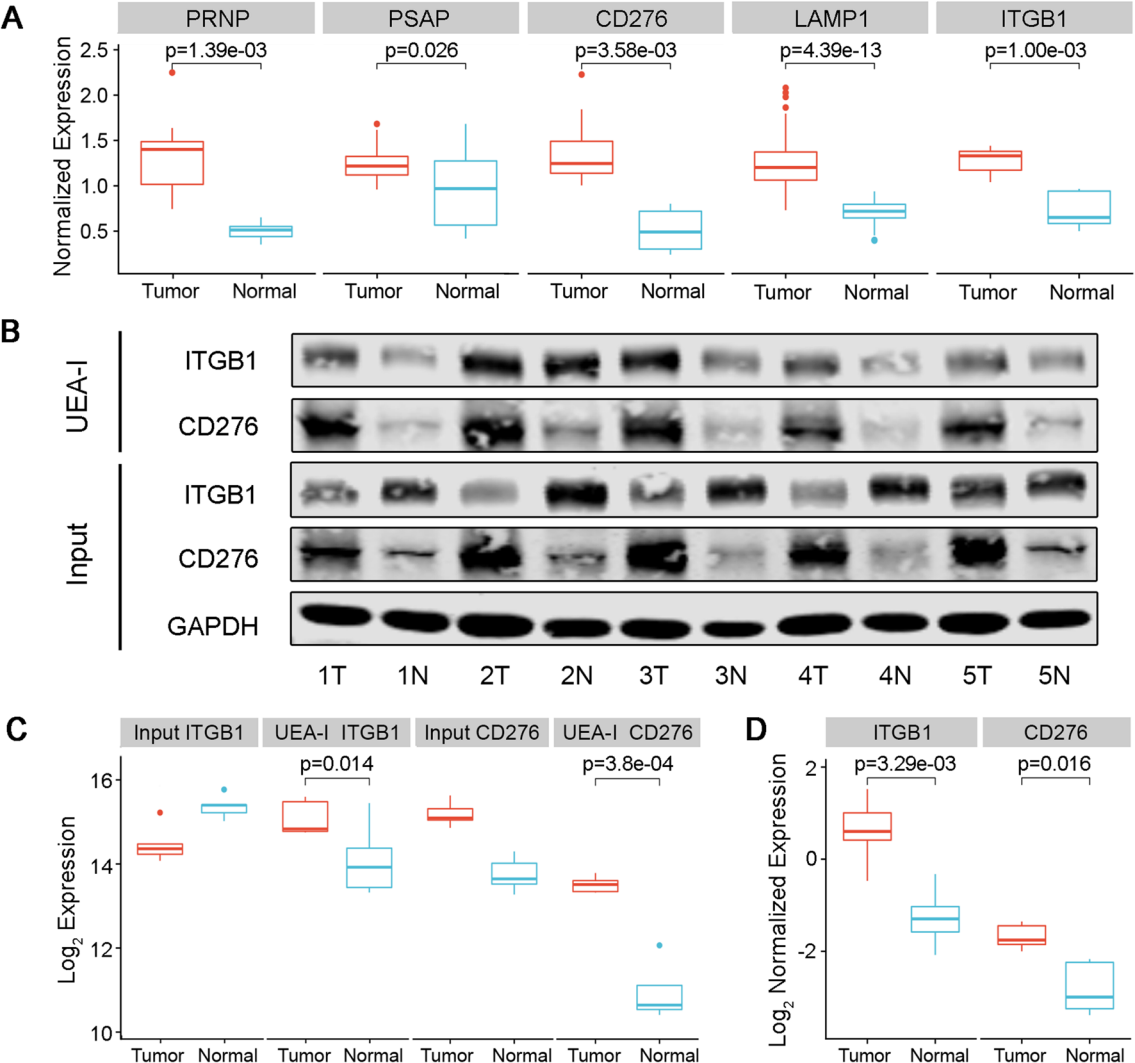
Validation of significantly altered glycoproteins in ESCC. **A** Normalized expression level of five significantly changed glycoproteins (PRNP, PSAP, CD276, LAMP1, ITGB1) in N-glycoproteomics. **B** UEA-I lectin affinity chromatography of ESCC tissue and paired normal tissue samples followed by western blot with ITGB1 or CD276 antibody. Input: total proteins extracted from tissues. UEA-I: fucosylated ITGB1/CD276 pulled down by UEA-I lectin affinity chromatography. **C** Gray-scale analysis results of **B** with log2 conversion. **D** Box plot of normalized fucosylated ITGB1 and CD276 glycoprotein expression levels

Next, we used the lectin affinity enrichment followed by western blot to further validate the biomarkers for ESCC. We chose PRNP, PSAP, CD276, LAMP1, ITGB1 in this phase based on the following considerations: (1) In the N-glycoproteomic site-mapping analysis at tissue level, they stood out as glycoproteins with significantly altered expression between ESCC and paired normal tissue (fold change > 1.5 after normalization by overall protein quantification, Fig. 7A); (2) The glycoproteins were identified in both N-glycoproteomics and lectin based mass spectrometry analysis at ESCC cellular level; (3) All the glycoproteins were identified in glycosite database; (4) All of them were predicted as secretory proteins by the program SignalP. The values predict a high likelihood of detecting the proteins remotely in the blood stream and may act as biomarkers in ESCC; (5) To the best of our knowledge, they have not been tested as glycoprotein markers for ESCC. UEA-I lectin enrichment followed by western blot showed significantly increased fucosylated levels of ITGB1 and CD276 in ESCC compared with paired normal tissue, while protein expression levels change to a lesser extent than fucosylation levels (Fig. 7B, C). Gray-scale analysis by ImageJ software also showed that the expression level of normalized fucosylated ITGB1 and CD276 in tumor tissues was significantly higher than those in normal tissues (Fig. 7D). The validation results indicated that elevated fucosylated ITGB1 and CD276 contributed to the occurrence and development of ESCC, which might be potential biomarkers in ESCC.

### Stage progression related dynamic clusters analysis in ESCC

As the lymph node status is one of the main factors affecting the clinical stage and survival of ESCC patients, we were interested in the dynamic process of glycoprotein expression during ESCC development, especially in lymph node metastasis (LNM). To investigate the types of dynamic change, we performed dynamic clusters analysis on the basis of glycoprotein quantity. We captured a series of glycoprotein clusters whose fold change increased or decreased with the progression of lymph node metastasis.

**Fig. 8:**
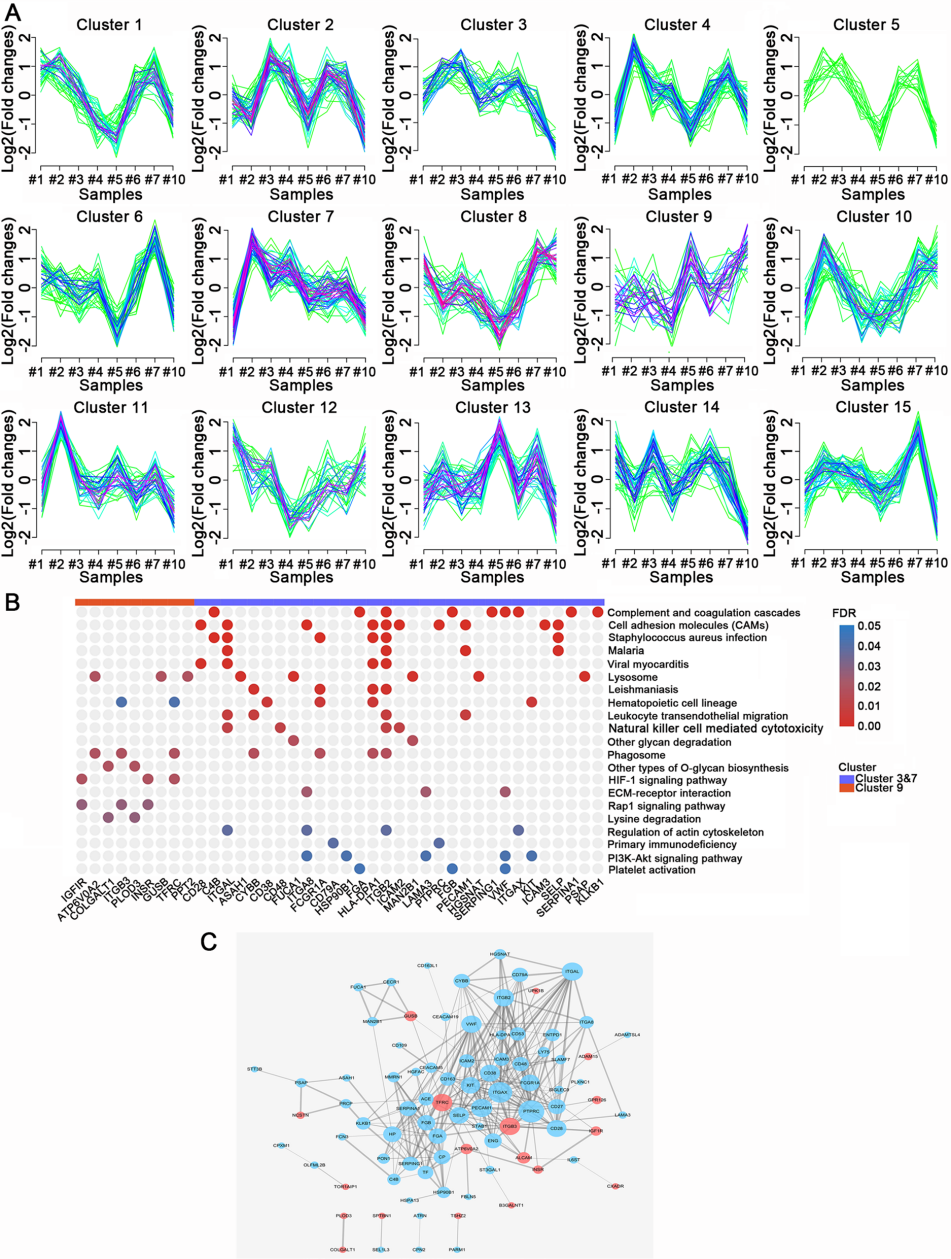
Enrichment-based dynamic cluster analysis of glycoprotein expression profile in ESCC development. **A** A total of 15 dynamic clusters analyses in the process of LNM. The abscissa represents the samples and the ordinate represents the quantitative values of each sample in log2 conversion. **B** KEGG enrichment analysis of increased and decreased fold-change glycoprotein dynamic clusters. **C** IPA analysis of the genes associated with cluster #3, #7 and #9

After further study about the dysregulated glycoprotein clusters in cancer progression, totally fifteen clusters were discovered in our data. Clusters #3, #7 and #9 represented a series of glycoproteins with expression change (Fig. 8A). Clusters #3 and #7 represented a series of glycoproteins whose fold-changes tend to decrease as the disease develops, while #9 was glycoprotein cluster with increasing fold-changes. The enrichment pathways and interaction networks of these glycoproteins were exhibited in Fig. 8B and C. The glycoproteins of Clusters #9 were enriched in HIF-1 signaling pathway (INSR, IGF1R, TFRC), RAP1 signaling pathway (INSR, IGF1R, ITGB3), and lysine degradation pathway (PLOD3 and COLGALT1), indicating that these pathways might play an important role in the LNM process in ESCC. Furthermore, the glycoproteins of Clusters #3 and #7 were enriched in cell adhesion molecules (CAMs), complement and coagulation cascades, and leukocyte trans-endothelial migration. The decreased glycosylation levels of CAMs may promote tumor cells to detach from the primary tumor and facilitate tumor metastasis. For leukocyte migration and complement system, low glycosylation levels may affect their immune surveillance and clearance capabilities of cancer cells.

## Discussion

Aberrant protein glycosylation is well known to be associated with the occurrence and development of cancer including cell transformation, invasion and metastasis [25]. Glycans are usually attached to proteins on the cell membrane or in the extracellular matrix. N-linked glycosylation are the addition of an oligosaccharide chain to an asparagine (Asn) residue within an amino acid sequence Asn-X-Ser/Thr (X should not be proline). O-linked glycosylation are the transfer of sugar chain to the oxygen atom of the serine(Ser), hydroxyl (Tyr) or threonine(Thr) residues [26]. Although some glycoproteins have been identified as potential biomarkers for various cancer types, the related studies and biomarkers in ESCC are scarce. This study identified a series of differentially expressed glycoproteins in ESCC which might serve as potential biomarkers for ESCC.

One of the highlights of this study was the normalization of N-glycoproteomics data by corresponding proteomics data. Different from our previous proteomic study of larger cohort [27], in this study, the TMT labeling technique was applied to one-to-one paired samples to exclude the possibility that the changes of N-glycosylation modification were caused by the protein abundance change from the same ESCC patient. Of course, some of the previous proteomic results have been confirmed in this smaller cohort. For example, ESCC were mainly characterized by elevated proteomic levels in the spliceosome, DNA replication and the cell cycle pathway.

As expected, the changes in many glycoproteins were accompanied by the changes in corresponding total protein levels. After normalization by protein expression, a total of 901 glycoproteins were identified and quantified. All the identified glycoproteins were mainly located in the subcellular compartments, consistent with distribution characteristics of the N-glycoprotein formation process. Moreover, the glycoproteins involved in the ECM-receptor interaction, focal adhesion (such as laminin, integrin and fibronectin family) and lysosome (e.g., LAMP1, GNS, LGMN, MAN2B1) were enriched which was consistent with the previous reports [19, 28]. These three pathways were also significantly enriched in our previous ESCC proteomics study [27], but accompanied by a significant decrease in the expression of related proteins, indicating the significant effect of glycosylation level on their function. Interestingly, the proteins involved in amino acid metabolism were both up-regulated and enriched. PLOD2, PLOD3 and PLOD1, which belong to lysyl hydroxylase and catalyze collagen lysine hydroxylation, were found to be up-regulated glycoproteins in all cases of our study. Although increased glycosylation of these proteins was also observed in colorectal cancer [29], the detailed mechanism of glycosylation in carcinogenesis remained un-elucidated. Glycosylation of HMGCS1 and AOC3 proteins have not been reported in any cancer types. HMGCS1 mediates the mevalonate pathway, ketogenesis [30] and is involved in cholesterol biosynthesis [31]. AOC3 catalyzes the conversion of primary amines into aldehydes [32]. The glycosylation level of AOC3 protein was up-regulated at Asn137 while the total protein level was down-regulated, indicating that an overall increase of N-glycosylation occupancy of AOC3 has an important impact on the biological function. All these results supported the role of glycosylation in cellular metabolism and ESCC progression [33].

The majority of differential glycoproteins with multiple glycosylation sites were laminin, integrin, collagen, and fibronectin associated proteins. Integrin beta-1 (ITGB1), a member of the integrin family, has been shown to play a crucial role in cell adhesion, migration and invasion in various cancers [34–36], whereas the effects of its glycosylation modifications on tumors have not been reported. Intriguingly, we found that ITGB1 protein expression levels were significantly lower in ESCC tumor tissues than in normal tissues, in accordance with previously reported data in ESCC proteomics [27], while the exact opposite was true for fucosylated ITGB1 levels. The expression level of fucosylated CD276 was similar to that of ITGB1 in our study. CD276, a highly glycosylated protein belonging to the immunoglobulin superfamily, has been implicated in tumor cell proliferation, migration, invasion, and angiogenesis, even acted as a dual role in the regulation of immune responses [37, 38]. Abnormal fucosylation of CD276 has been reported in triple negative breast cancer (TNBC) and oral squamous cell carcinoma, and can inhibit the immune response in TNBC [39, 40]. Our study bodes well for the abnormal fucosylation of ITGB1 and CD276 as new prospective diagnostic and therapeutic features of ESCC. However, the specific molecular mechanism of their effects on the occurrence and development of ESCC remains to be further studied.

Among other identified glycoproteins, lysosome-associated membrane glycoprotein 1 (LAMP1) has been reported to be a pro-invasive factor in cancer progression through abnormal localization on the plasma membrane of cancer cells [41]. This may be a membrane repair mechanism formed by lysosomal fusion and extracellular interaction [42]. On the other hand, LAMP1 localization on the plasma membrane provided the binding ability to E-selectin through sialyl-LeX residues, thereby promoting the cancer cells adhering to extracellular matrix [43]. Over-expressed LAMP1 also can influence cancer development inside the lysosomal membrane through increasing lysosomal exocytosis and lysosomal size [44, 45]. In this study, N-glycosylation level of LAMP1 was up-regulated but the protein expression level was not changed. This indicated that the glycosylation modification but not expression change might be involved in ESCC development. Whether glycosylation of LAMP1 affects localization and how the six differential glycosylation sites of LAMP1 (Asn84, 103, 261, 76, 62, 249) in ESCC needs further investigation. Transferrin receptors encoded by TFRC is a membrane glycoprotein, which can import iron by binding transferrin. TFRC displayed moderate to strong cytoplasmic expression in various cancer tissues. The expression level is consistent with tumor stages [46]. TFRC knockdown decreased intracellular total iron, suppressed tumor growth and metastasis in human and mouse mammary adenocarcinomas [46]. Matrix remodeling associated 5 (MXRA5) was a novel extracellular protein that was also up-regulated in several types of cancers [47]. MXRA5 was identified to be frequently mutated in non-small cell lung carcinoma and the high MXRA5 expression was correlated with tumor progression [48, 49]. However, no report has elucidated the role of the N-glycosylation modification of this protein in any cancer type.

Fifteen glycoprotein clusters with fold change in Lymphatic metastasis (LNM) progresses were identified by dynamic analysis. And the glycoproteins that increased with LNM status were enriched in HIF-1 signaling pathway, RAP1 signaling pathway and lysine degradation pathway. HIF-1 signaling is a classical oncogenic pathway associated with tumor growth, angiogenesis, metastasis, and mortality. Recent studies showed HIF-1 signaling was involved in lymphatic invasion through induction of platelet-derived growth factor B (PDGF-B) in breast cancer [50], vascular endothelial growth factor C (VEGF-C) [51] and SP1 in ESCC [52]. INSR and IGF1R belong to Insulin/IGF System, which was known to affect the malignant behavior of cancer cells and was regulated by Glycans [53]. Increased INSR/IGF1R were correlated with LNM in cancers [54]. Inhibition of N-linked glycosylation impaired the glycosylation of the receptors (INSR and IGF1R) and reduced their abundance at the cell surface [55]. As an important cell adhesion molecular, ITGB3 expression has been reported to be associated with LNM in several types of cancers [56]. N-glycosylation was required for cell attachment to ECM [57]. RAP1 pathway is also an important regulator of cell adhesions and junctions, cellular migration, and polarization [58]. RAP1 expression was reported to be correlated with LNM in ESCC and its knockdown decreased the migratory and invasive capacities via AKT signaling in ESCC cells [59]. The role of lysine degradation pathway in cancer was rarely reported. In colorectal cancer, thrombopoietin (TPO) was known to promote self-renewal and metastasis of CD110+ tumor-initiating cells (TICs) to the liver by activating lysine degradation [60]. Here, we found two genes involved in lysine degradation, PLOD3 and COLGALT1, were involved in collagen glycosylation. PLOD3 was up-regulated in some types of cancers and promoted tumor malignant progression [61–65]. ICOLGALT2 was also reported to be overexpressed in metastatic ovarian cancer and interacted with PLOD3 [66].

Our study fills a gap in the field of glycoproteomics research in ESCC to some extent. However, how the abnormal glycosylation modifications and glycoprotein biomarkers identified in this study affect the occurrence and development of ESCC needs to be further explored.

## Conclusion

Our study revealed a series of differentially expressed glycoproteins and signaling pathways in ESCC. Several metastasis-related glycoproteins and signaling pathways were also identified. The results might provide some potential biomarkers and therapeutic targets for the diagnosis and treatment of ESCC patients.

## Supplementary Information

**Additional file 1: Table S1.** Clinical features of the ESCC patients cohort. **Table S2.** Differentially expressed proteins in proteomics (fold change > 1.5 and p value < 0.05). **Table S3.** Motif logo and model. **Table S4.** Differentially expressed glycoproteins and glycosylation sites in normalized N-glycoproteomics (fold change > 1.5 and p value < 0.05). **Table S5.** The details of the 179 significantly regulated glycoproteins in ESCC. **Table S6.** Label-free based N-glycoproteomics of ESCC cell line normalized by proteomes. **Table S7.** LCA enriched glycoproteins in ESCC cell line. **Table S8.** UEA-I enriched glycoproteins in ESCC cell line.

## Abbreviations

ESCC: Esophageal squamous cell carcinoma
SCC: Squamous cell carcinoma
CEA: Carcinoembryonic antigen
CYFRA21-1: Cytokeratin-19-fragment
Fut-Hpt: Fucosylated haptoglobin
MS/MS: Tandem mass spectrometry
AGC: Automatic gain control
FDR: False positive rate
GO: Gene Ontology
KEGG: Kyoto Encyclopedia of Genes and Genomes
FCM: Fuzzy c-means algorithm
PTM: Post-transcriptional modification
PLOD2: Procollagen-lysine,2-oxoglutarate 5-dioxygenase 2
COL6A3: Collagen alpha-3(VI) chain
FKBP10: Peptidyl-prolyl cis–trans isomerase
TNC: Tenascin
PLOD1: Procollagen-lysine,2-oxoglutarate 5-dioxygenase 1
PLOD3: Procollagen-lysine,2-oxoglutarate 5-dioxygenase 3
ACO3: Amine oxidase copper containing 3
IL4I1: Interleukin 4 induced 1
HMGCS1: 3-Hydroxy-3-methylglutaryl- CoA synthase 1
LCA: Lens culinaris agglutinin
UEA-I: Ulex europaeus agglutinin 1
ITGB1: Integrin beta-1
CD276: CD276 molecule
LNM: Lymph node metastasis
CAMs: Cell adhesion molecules
MXRA5: Matrix remodeling associated 5
LAMP1: Lysosome-associated membrane glycoprotein 1
TFRC: Transferrin receptor protein 1
PDGF-B: Platelet-derived growth factor B
VEGF-C: Vascular endothelial growth factor C
TICs: Tumor-initiating cells (TICs)
